# Metastatic behaviour of primary human tumours in a zebrafish xenotransplantation model

**DOI:** 10.1186/1471-2407-9-128

**Published:** 2009-04-28

**Authors:** Ines J Marques, Frank Ulrich Weiss, Danielle H Vlecken, Claudia Nitsche, Jeroen Bakkers, Anne K Lagendijk, Lars Ivo Partecke, Claus-Dieter Heidecke, Markus M Lerch, Christoph P Bagowski

**Affiliations:** 1 Institute of Biology, Department of Integrative Zoology, University of Leiden, 2333 AL, Leiden, The Netherlands; 2 Universitätsklinikum Greifswald, Klinik für Innere Medizin A, D-17475, Greifswald, Germany; 3 Hubrecht Institute & University Medical Centre Utrecht & Interuniversity Cardiology Institute of the Netherlands, 3584 CT, Utrecht, The Netherlands; 4 Universitätsklinikum Greifswald Abteilung für Allgemein, Viszeral, Thorax und Gefäßchirurgie, D-17475, Greifswald, Germany

## Abstract

**Background:**

Aberrant regulation of cell migration drives progression of many diseases, including cancer cell invasion and metastasis formation. Analysis of tumour invasion and metastasis in living organisms to date is cumbersome and involves difficult and time consuming investigative techniques. For primary human tumours we establish here a simple, fast, sensitive and cost-effective *in vivo *model to analyse tumour invasion and metastatic behaviour.

**Methods:**

We fluorescently labelled small explants from gastrointestinal human tumours and investigated their metastatic behaviour after transplantation into zebrafish embryos and larvae. The transparency of the zebrafish embryos allows to follow invasion, migration and micrometastasis formation in real-time. High resolution imaging was achieved through laser scanning confocal microscopy of live zebrafish.

**Results:**

In the transparent zebrafish embryos invasion, circulation of tumour cells in blood vessels, migration and micrometastasis formation can be followed in real-time. Xenografts of primary human tumours showed invasiveness and micrometastasis formation within 24 hours after transplantation, which was absent when non-tumour tissue was implanted. Furthermore, primary human tumour cells, when organotopically implanted in the zebrafish liver, demonstrated invasiveness and metastatic behaviour, whereas primary control cells remained in the liver. Pancreatic tumour cells showed no metastatic behaviour when injected into cloche mutant embryos, which lack a functional vasculature.

**Conclusion:**

Our results show that the zebrafish is a useful *in vivo *animal model for rapid analysis of invasion and metastatic behaviour of primary human tumour specimen.

## Background

Approximately 90% of all cancer deaths arise from the metastatic spread of primary tumours [1]. Metastasis formation is a complex, multi-step process in which primary tumour cells invade neighbouring tissues, enter the systemic circulation (intravasate), translocate through the vasculature, arrest in distant capillaries, extravasate into the perivascular tissue, and finally proliferate from micrometastases into macroscopic secondary tumours [2]. Invasiveness and early formation of metastases are the main reasons why for example pancreatic cancer continues to have a dismal prognosis, with a 5 year survival rate of <5% and a mean life expectancy of <6 month [1].

Zebrafish and their transparent embryos have been employed in several useful models for therapeutic drug research and preclinical studies [3]. High throughput screening (HTS) in zebrafish embryos has been established and is nowadays commonly used for different applications [3-5]. A number of unique features make this animal model very attractive: zebrafish are inexpensive to maintain, breed in large numbers, develop rapidly *ex vivo*, and can be maintained in small volumes of water [6]. Recently, the zebrafish and its transparent embryos have also come into view as a new model system to investigate tumour development, cancer cell invasion and metastasis formation [7-11]. Mary Hendrix and her group have pioneered the field of cancer cell transplantation in zebrafish embryos and could show that transplanted human malignant melanoma cells are not rejected, survive and even exhibited motility [12,13]. Haldi et al. observed the formation of tumour-like cell masses when xenotransplanting human melanoma cells in slightly older zebrafish embryos [14]. Several independent studies have now shown that human melanoma cells and other cancer cell lines are able to induce neovascularization when xenografted in the zebrafish [14,11,16].

The role of the small GTPase RhoC in tumour formation, angiogenesis and cell invasion was investigated in real-time in 1-month-old immunosuppressed zebrafish xenografted with the human breast cancer cell line MDA-435 [11]. This study achieved high-resolution imaging of the dynamic cell-vascular interface in transparent juvenile zebrafish. All these innovative studies established the use of the zebrafish xenotransplantation model for the analysis of cancer cell lines. In this study we now show that zebrafish embryos can even be used to directly transplant human tumour tissue and primary human tumour cells. Zebrafish embryos thus provide a simple, fast and cost-effective method to test the metastatic behaviour of primary tumours in an *in vivo *vertebrate animal model that also permits high throughput drug screening.

## Methods

### Animal care and handling

Zebrafish (*Danio rerio*) (Tuebingen line, alb strain (Albinos) and Tg(fli1:eGFP) were handled in compliance with local animal care regulations and standard protocols of the Netherlands and Germany. Fish were kept at 28°C in aquaria with day/night light cycles (10 h dark versus 14 h light periods). The developing embryos were kept in an incubator at constant temperatures. The cloche (clo) mutant line has been previously described [17]. Heterozygous fish (clo^-/+^) are kept and bred under normal conditions. 25% of offspring will consist of homozygous clo^-/- ^mutants which lack functional vasculature and circulation 75% will be siblings with no phenotype. Lack of circulation, an enlarged pericardium and curvature of the tail (at a later time point) are hallmarks of the cloche phenotype.

### Cell culture

EpRas cells were cultured at 37°C in DMEM high glucose containing L-glutamine, 4% FCS and 1:100 Pen/Strep (GIBCO, Invitrogen). PaTu8988T and PaTu8898S cells were cultured in DMEM high glucose, with 10% FCS and 1:100 Pen/Strep. The EpRas cells were treated with recombinant human TGF-β1(RD systems) at a final concentration of 2 ng/ml. To induce epithelial to mesenchymal transition (EMT), cells were seeded at 70% confluency in 6-well plates and media containing TGF-β1 (2 ng/ml final concentration) was added and replaced every other day for 10 days. After this period, cells were ready for injection.

### Cell staining, injections and incubations

Cells were stained with either CM-Dil (red fluorescence) or DiO (green fluorescence) (Vybrant, Invitrogen). Cells were seeded in 6-well plates, grown to confluency trypsinized (without EDTA for EpRas cells or with EDTA for all other cells used). Subsequently, cells were washed with 67% DPBS (GIBCO, Invitrogen), transferred to 1.5 ml Eppendorf tubes and centrifuged 5 min, at 1500 rpm. Cells were re-suspended in DPBS containing either CM-Dil (4 ng/ul final concentration) or DiO (200 μM final concentration). Cells stained with CM-Dil were incubated 4 min at 37°C and then 15 min at 4°C. Cells stained with DiO were incubated 20 min at 37°C. After this period cells were centrifuged 5 min at 1500 rpm, the supernatant discarded and cells re-suspended in 100% FCS, centrifuged again and washed 2 times with 67% DPBS. Cells were suspended in 67% DPBS for injection into the embryos. 2 dpf zebrafish embryos were dechorionated and anesthesized with tricaine (Sigma). Using a manual injector (Eppendorf; Injectman NI2), the cell suspension was loaded into an injection needle (15 μm internal- and 18 μm external-diameter). Cells were now injected in 2 dpf albino or Tg(fli1:eGFP) zebrafish embryos. After injection, embryos were incubated for 1 h at 31°C and checked for cell presence at 2 hpt. Fish with fluorescent cells outside the implantation area at 2 hpt were excluded from further analysis. All other fish were incubated at 35°C for the following days.

### Tissue preparation for transplantation into zebrafish embryos

Human material from surgical resection specimens was obtained at the Universitätsklinikum Greifswald according to local ethical guidelines and after obtaining informed patient consent. Tumour tissue and control tissue were cut into very small pieces using a scalp blade. A piece of tissue was then transferred to a 2 ml Eppendorf tube, washed with 67% DPBS and stained with 1:500 CM-Dil. The tissue was incubated for 6 min at 37°C and 20 min at 4°C. Washing procedures were the same as mentioned above for the cells. Before transplantation small pieces of stained tissue were further disaggregated using Dumont forceps (No.5) into a relative size of 1/5 to 1/2 the size of the yolk. Tissue pieces with the correct size were transferred to agarose plates in which the embryos were laying, ready for transplantation. For tumour and control transplantations, a glass transplantation needle was used to transfer the tissue into the yolk. With the glass transplantation needle a piece of tissue was picked up, put on top of the yolk and then pushed inside. The yolk usually sealed itself and in the majority of embryos, the tumour remained in the yolk. After transplantation, embryos were incubated for 1 h at 31°C, then embryos were checked for presence of tissue and incubated at 35°C for the following days.

### Cell dissociation from tissue

Tissue samples were cut in very small pieces using a scalp blade. Cut tissue pieces were then transferred to 6 ml glass containers with 3 ml isolation media (180 ml DMEM high glucose, 20 ml 100 mM HEPES, 46 ml 5% BSA) and Collagenase (Invitrogen) (50 μl of a 6 mg/ml stock solution for each 12 ml of isolation media). Tissue was incubated in a water bath for 15 min at 37°C. The supernatant was decanted and tissue pieces were cut further into smaller pieces using a scalp blade. Tissue pieces were again incubated 15 min at 37°C in 3 ml isolation media with collagenase. Afterwards tissue pieces were transferred to 15 ml falcon tubes and cells were dissolved by pipetting up and down through serial cut blue pipette tips (5 different diameters). The cell suspension was now filtered through 2 sheets of gaze, into 2 ml Eppendorf tubes and centrifuged 5 min at 1500 rpm. The supernatant was discarded and cells re-suspended in isolation media. The described procedure was then repeated once. For injections, cells were stained with either CM-Dil or DiO and injected into 2 dpf zebrafish embryos, as described above.

### Western-Blotting

Pancreatic cancer cell lines PaTu-S and PaTu-T were lysed in iced Triton-X-100 lysis buffer (0.1%) containing protease inhibitors (1 ml/mg tissue, 10 μg/ml aprotinin, 10 μg/ml leupeptin, 0,01 M sodiumpyrophosphate, 0,1 M sodiumfluoride, 1 mM dihydrogenperoxide, 1 mM L-phenyl-methyl-sulfonyl-fluoride [PMSF] and 0,02% soybean-trypsininhibitor). Protein concentration was determined by a modified Bradford-assay (Bio Rad Laboratories, München, Germany) and equal amounts of protein were used in subsequent experiments. Cell lysates were separated by SDS-PAGE on a 7.5% polyacrylamide gel in a discontinuous buffer system and gels were blotted on nitrocellulose membranes (Hybond C, GE Healthcare Europe GmbH). After overnight blocking in NET-gelatine (10 mM Tris/HCl pH 8.0, 0.15 mM NaCl, 0.05% TWEEN 20, 0.2% gelatine) immunoblot analysis was performed followed by enhanced chemoluminescence detection (GE Healthcare Europe GmbH) using horseradish peroxidase coupled sheep anti-mouse IgG or goat anti-rabbit IgG GE Healthcare Europe GmbH). Monoclonal E-cadherin antibody (Clone 36), directed against the carboxy-terminus, was purchased from Transduction Laboratories (San Diego, CA, USA) as well as antibodies against α-, β-, and γ-Catenin. A polyclonal G3PDH antibody was purchased from Biozol (Eching, Germany).

### Immunofluorescence microscopy

PaTu-S and PaTu-T cells grown on glass coverslips for 24–48 h were washed 3 times with PBS, fixed for 15 min in 4% paraformaldehyde and permeabilised in 0.1% Triton- X-100 for 5 min. Blocking of unspecific binding was achieved by a 1 h incubation in 10% Aurion BSA-c (Aurion, Waageningen, The Netherlands). Following a primary antibody incubation over night (dilutions 1:100) and subsequent PBS washing steps detection was performed using dichlortriacinyl aminofluoresceine (DTAF) or Cy3-coupled sheep IgG (dilutions 1:200). Nuclei were stained by a 30 sec. incubation with DAPI (1:10000 in PBS). After a final washing step in PBS cell were mounted in Vectashield (Vector Labs, Burlingame, CA, USA). Microscopic Images were taken using an AxioCam digital microscope camera on a Zeiss Axiophot microscope.

### *In vitro *migration assay ("scratch"-assay)

The scratch-assay was performed as previously described by Liang et al. [18]. Cells were grown to confluency in 6-well dishes and mitomycin C was added at 10 μM for 2 h. Then the cell monolayer was scraped in a straight line with a 200 μl pipette tip. Pictures of the scratch were taken under an invert Olympus microscope at 0 h, 12 h and 24 h.

### Histology of zebrafish embryos

Transversal sections at 4 μM thickness were prepared as described before [19]. Coupes were directly imaged with fluorescence microscopy or differential interference (DIC) microscopy. After fluorescent pictures were taken, Hematoxylin/Eosin (HE) staining was performed as described earlier [20].

### Whole mount immunofluorescence of zebrafish embryos

Zebrafish embryos at 2 dpt were fixed overnight in 4%PFA in PBS at 4°C. After fixing, embryos were washed with BSAc 0.1%-TritonX100 1% in PBS (blocking buffer; 3 × 10 minutes). Subsequently, the embryos were incubated for 2 hrs in blocking buffer at RT. Incubation with the primary antibody (Mouse anti-Proliferating Cell Nuclear Antigen, PCNA, from Zymed Laboratories, 1:100) was done overnight at 4°C. After washing (3 × 10 minutes) with blocking buffer, embryos were incubated with the secondary antibody (Fluorescein (DTAF)-conjugated AffiniPure Goat anti-Mouse IgG, Jackson Immuno Research Laboratories, Inc., 1:100) for 1 hr. and washed afterwards with blocking buffer (3 × 10 minutes). Embryos were mounted in 3% methylcellulose to orient them properly for imaging. Imaging was done with confocal scanning laser microscopy (Biorad 1024ES; Software: Biorad Laser sharp 2000).

### Imaging, selection and positioning of transplanted zebrafish embryos

Confocal pictures were taken either with the Biorad Confocal microscope 1024ES (Zeiss microscope) combined with Krypton/Argon laser, or the dual laser scanning confocal microscope Leica DM IRBE (Leica) or with the Nikon TE300 confocal microscope and a coherent Innova 70C laser (Chromaphor, Duisburg, Germany). Pictures were further taken by DIC microscopy using the Axioplan 2 microscope with an AxioCam MR5 camera (Carl Zeiss). Further, fluorescent stereomicroscope pictures were taken with the Leica DFC 420C camera attached to a Leica MZ16FA microscope. Two hours post implantation the embryos were anesthetized with tricaine and positioned laterally, with the site of the implantation to the top, on 3% methylcelulose, on a slide with depression. Each time two rows of twenty embryos were screened. Two hours post implantation every embryo that showed cells outside the area of implantation was discarded and not considered for the experiment.

## Results

### Tumour cell xenografts in zebrafish embryos

**Figure 1:**
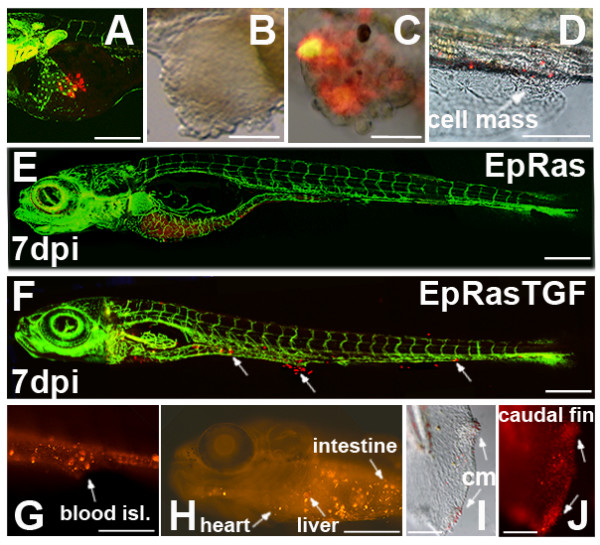
**Migration and cell mass formation of Ha-Ras transformed mouse mammary epithelial cells injected into the yolk sac of zebrafish embryos**. EpRas (parental) and EpRas cells stimulated with TGF-β (EpRasTGF) were labelled and ectopically injected into the yolk sac of 2 dpf zebrafish embryos. In A, E and F transgenic zebrafish embryos expressing GFP under an endothelial promotor (Tg(fli1:eGFP) were used. An example of newly injected EpRas cells at 1 hour post injection is given in (A). In (B) an ectopic tumour cell mass formed in the yolk sac by EpRasTGF cells is shown. Examples of cell masses formed by EpRasTGF cells at distance from the place of injection are shown for the tail region (C) and blood islands with surrounding ventral fin (D). Pictures in B-D were taken at 3 dpi. While EpRas cells remained in the yolk and never invaded the embryo (E), EpRasTGF cells invaded, migrated and formed distant micrometastases, which are indicated with arrows (F). Red fluorescence of cells is still visible after 7 dpi (E, F). Images G to J show tumour cell masses (cm) and migrated cells in blood islands (blood isl.), the liver, heart, intestine and the caudal fin of 6 dpi larvae. Scales shown are for A: 200 μm; D-H: 600 μm, for B, C, I and J: 100 μm. 3D reconstructions of EpRAS and EpRasTGF cells in zebrafish larvae are shown in two supplemental movies (see Additional file 2 and Additional file 3).

Mouse mammary epithelial cells (EpH4) transformed with oncogenic Ras (EpRas) have been used to establish a mouse tumourigenesis model over a decade ago [21]. In these EpRas cells, TGF-β signalling causes epithelial to mesenchymal transition (EMT) which transforms cells to a highly invasive phenotype and enables distant metastasis formation when transplanted into nude mice [22]. Initially, we evaluated metastasis formation using this well-characterized system in the zebrafish cell xenograft model. We transplanted fluorescently labelled EpRas cells into the yolk sac of 2 day old zebrafish embryos to study metastatic behaviour *in vivo*. EpRas cells that had been stimulated with TGF-β for 10 days prior to injection, showed metastatic behaviour in the zebrafish, comparable to results previously reported in mice [21-23]. Following EMT the cells invaded embryonic tissue, entered the circulation and homed in at distant tissues and organs. EpRas TGFβ treated cells were found in blood islands, brain, caudal fin, caudal vein, gill arches, heart, intestine, liver, mandible, optic cup (eye), otic cup, pericardium, somites, swim bladder. However, they had a tendency to invade and home in to muscle tissue, head structures, caudal fin and blood islands (Fig. 1 and Additional File 1). To a lesser extent, we observed invasion of these cells into the liver or other organs of the gastrointestinal tract. In contrast, unstimulated EpRas control cells remained at the place of injection in the yolk and neither invaded the developing zebrafish nor did they enter blood circulation (Fig. 1 and Additional file 2). In three independent experiments the average percentage of migrating cells observed for the EpRas TGF-β-stimulated cells was 46.6% (SD +/- 2.0; p-value < 0.001) compared to 0.5% (SD +/- 0.7; p-value < 0.001) for the parental EpRas cells (see Additional file 1). Furthermore, the TGF-β stimulated EpRas cells formed tumour cell masses in the developing zebrafish (Fig. 1), which resemble the formation of metastases in nude mice[23]. In the zebrafish, cells begin to invade the embryo already several hours after injection (on average 4 hours post injection)(see Additional File 3) and tumour cell masses are visible as early as 3 days post implantation (dpi).

For optimal visualization, we used the transgenic zebrafish line, Tg(fli1:eGFP)[11,24], which expresses GFP under the *fli1 *promotor (an early endothelial marker) and therefore exhibits a green fluorescent vasculature [11,24]. In a time lapse movie (see Additional file 4; rate: 1 frame/minute) we show an example of fluorescently labelled EpRas TGF-β cells (3 dpi) which have invaded the zebrafish body, have translocated into the vasculature and have colonized at distant sites in the zebrafish larvae (5 dpf). Some cells are visible in the blood stream whereas others have extravasated from the vasculature. Evaluation of metastasis formation in the zebrafish model is therefore significantly faster than in currently used mouse models, where it may take several weeks until metastases become detectable [23]. The sensitivity of the zebrafish tumour xenograft model further allows observation of individual cells and their daughter cells *in vivo*.

**Figure 2:**
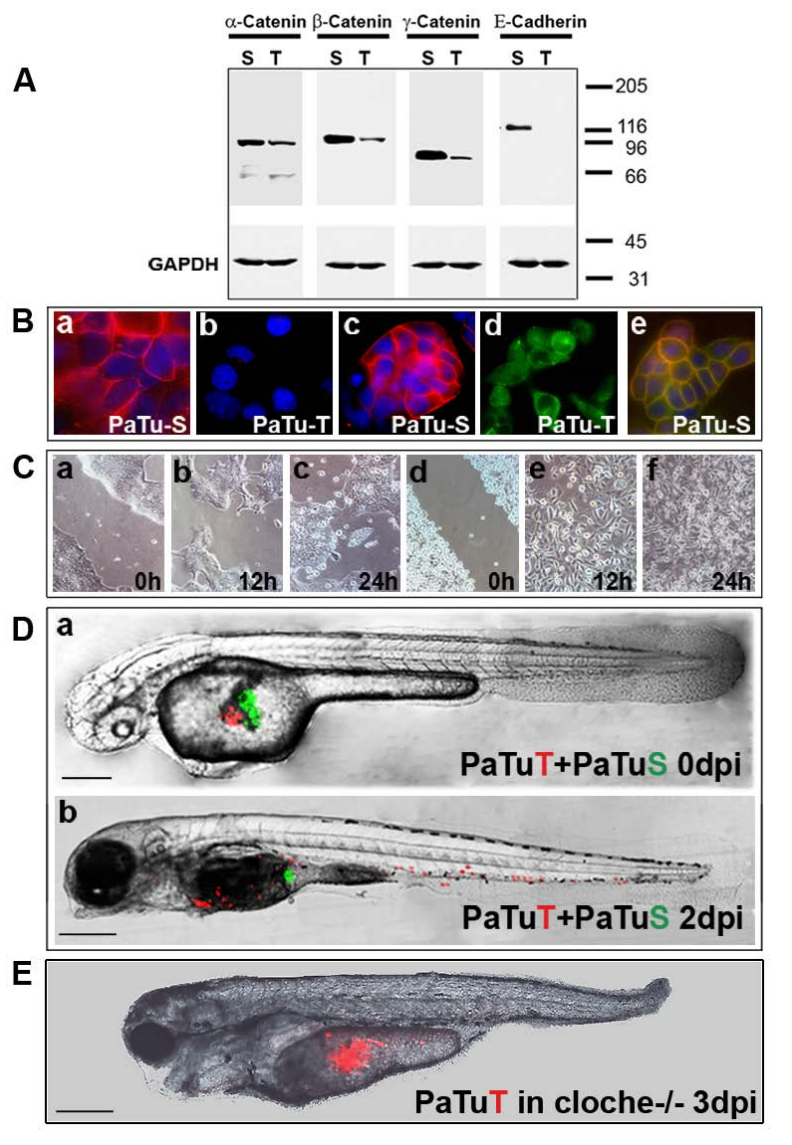
**Implantation of two pancreatic cancer cell lines into the same zebrafish embryo**. **(A**) Western blot analysis shows that PaTu-S but not PaTu-T cells express E-cadherin and both express α-, β- and γ-catenin. GAPDH expression is shown as a control. (**B**) Cellular localization of E-cadherin and β-catenin was analysed by immunofluoresscence. E-cadherin expression is shown for PaTu-S cells (a) and absence of E-cadherin expression for PaTu-T cells (b). Dapi staining was used to visualize cell nuclei in blue. β-catenin localization is shown for PaTu-S (c) and for PaTu-T (d). Co-localization of E-cadherin (green) and β-catenin (red) in PaTu-S cells is indicated by yellow staining of the plasmamembrane (e). (**C**) An *in vitro *migration assay ('scratch assay') shows differences in migration of the two cell lines (PaTu-S: a-c and PaTu-T: d-f). Similar results were obtained in four independent experiments. Gap closure (gap width) over time is shown in Additional file 5. (**D**) Non-invasive PaTu-S cells (green) and invasive PaTu-T cells (red) were implanted consecutively in the same embryo (a and b) (Scales: 250 μm (a) and 300 μm (b)). (**E**) Homozygous *cloche *mutants [17] were injected with PaTu-T cells and followed over time. Shown is an example of a cloche^-^/^- ^zebrafish at 3 dpi (scale bar: 300 μm). In contrast to control zebrafish none of the tested cloche^-^/^-^mutants showed any sign of metastatic behaviour (see Additional file 1 and Additional file 7). The cloche phenotype and its lack of a functional vasculature and circulation is observable by DIC microscopy (see Additional file 6).

We also compared the two established human pancreatic tumour cell lines, PaTu8988-S and PaTu8988-T [25] (referred to herein as PaTu-S and PaTu-T) in their invasive and metastatic potential in a single zebrafish. Both sister cell lines originate from liver metastases of the same human pancreatic adenocarcinoma [25]. E-Cadherin expression in PaTu-S cells (Fig. 2A and 2B(a)) correlates with the maintenance of functional cell-cell contacts (Fig. 2B) and a reduced tendency of cells to migrate (Fig. 2C)[26,27]. Whereas PaTu-S cells show localization of E-cadherin/β-catenin complexes at the plasmamembrane (Fig. 2B(e)), PaTu-T cells lack E-Cadherin expression (Fig. 2A and 2B(b)) and β-catenin is mainly localized in the cytoplasm (Fig. 2B(d). This observation is paralleled by enhanced migratory capabilities of PaTu-T cells compared to PaTu-S cells, which we confirmed in an *in vitro *migration assay ('scratch assay' [28] (Fig. 2C and Additional file 5).

We then labelled PaTu-S cells with green fluorescence and PaTu-T cells with red fluorescence. When implanted successively in the yolk sac of the same zebrafish embryo, green PaTu-S cells remained in the yolk, whereas red PaTu-T cells displayed invasion and metastatic behaviour (Fig. 2D and Additional file 1). Similar results were observed when cells were mixed prior to injections (data not shown).

PaTuT cells were found in the brain, caudal vein, gill arches, gut, heart, intestine, liver, operculum, pericardium, somites, swim bladder. They showed a tendency to invade and home in to organs of the gastrointestinal tract. Micrometastasis was often observed in organs such as the liver, the gut and the intestine. Invasion and homing in of these cells into muscle tissue was observed to a lesser extent. This behaviour was qualitative different from what we observed for TGF-β treated EpRas cells. We further tested the metastatic behaviour of PaTu-T cells in homozygous *cloche *mutants (cloche^-^/^-^), which lack a functional vasculature and circulation ([17] Additional File 6). In contrast to control zebrafish, no metastatic behaviour was observed in the cloche^-^/^- ^fish, indicating that the observed invasion/migration of PaTu-T cells indeed involves metastasis formation through the vascular system. Zebrafish were followed until three days post injections (Fig. 2E; see Additional file 1 and see Additional file 7).

### Human tumours transplanted into zebrafish display metastatic behaviour

We then pursued our primary goal to employ the zebrafish also as a simple, fast and effective test system for metastasis formation of primary human tumours. After informed patient consent small fragments of tumour explants from pancreas, colon and stomach carcinoma, as well as tumour-free areas from the same resection specimen were fluorescently labelled with CM-DIL and directly xenotransplanted into the yolk sac of zebrafish embryos. Tumour and non-tumour control cells were followed live by laser scanning confocal microscopy. Tumour cells started to invade the embryo on average 12 hours post injection and micrometastasis formation was visible as early as 24 h post injection. In parallel, we also investigated and compared the invasive and metastatic behaviour of tumour cells that had been dissociated from primary human tumours by collagenase digestion prior to transplantation.

**Figure 3:**
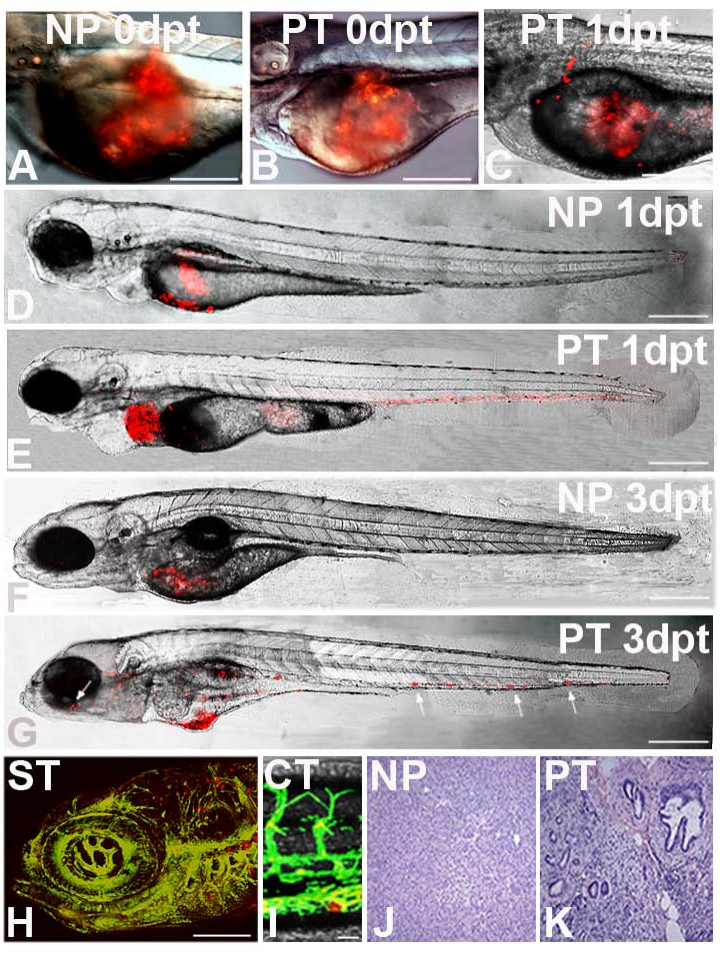
**Tumour transplantation in zebrafish**. Primary human tumours of the pancreas, the stomach and the colon were transplanted into 2 dpf embryos. Non-tumour tissue was used as control. At the respective time points indicated laser confocal microscopy images were taken. Images A and B show newly transplanted embryos with normal pancreas (NP) and pancreatic tumour (PT) respectively. Image C shows an example of an embryo transplanted with an adenocarcinoma of the pancreas at 1 day post transplantation (dpt) in which tumour cells have already invaded the embryo. Images D to G are confocal microscopy images of transplanted embryos at 1 dpt and 3 dpt. Normal, non-transformed pancreas transplants remain in the yolk and cells never migrate or spread in the embryo (D and F). In contrast, tumour transplants show metastatic behaviour (E and G). Some of the cell masses are marked with arrows, including one formed near the retina of the eye (G). On the bottom an example is shown for brain metastases of a transplanted gastric cancer (stomach tumour) in a Tg(fli1:eGFP) zebrafish 3 days after implantation(H). Cell masses are visible in the rhombencephalon (hindbrain) surrounding the otic capsule and near the gill arches (H). A colon tumour transplant shows a migrated tumour cell in the caudal vein region at 3 dpt (I). Both pictures (H and I) were taken by confocal microscopy. HE staining of representative histological sections of normal human pancreas tissue (J) and pancreatic cancer (K) are shown. Scales shown are in A-E: 300 μm; F, G: 400 μm; in H: 100 μm and in I: 20 μm.

**Figure 4:**
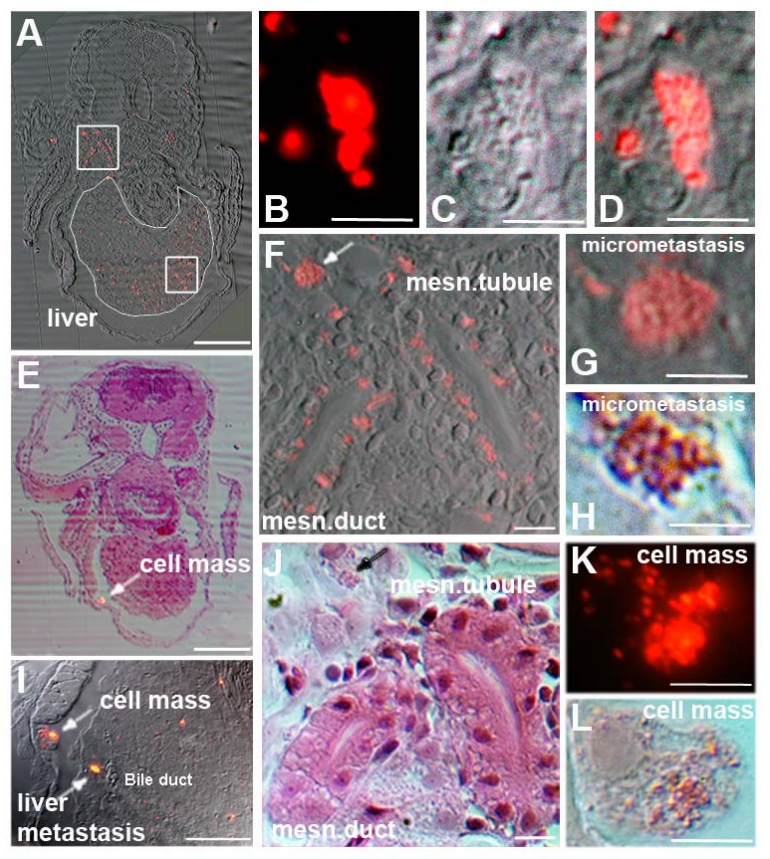
**Histology of zebrafish embryos transplanted with a human pancreatic tumour**. Transversal sections of zebrafish embryos transplanted with a primary human pancreatic tumour show the presence of micrometastases in different tissues at 3 dpt in 5 day old zebrafish. (A) The transversal section is approximately 40 μm caudal to the anterior end of the liver. The liver is circled with a thin white line and contains many tumour cells and some micrometastases. The square in the liver contains several micrometastases, of which one is depicted in higher magnification in B (fluorescence), C (DIC) and D (overlay). The upper square shows tumour cells and micrometastases around the mesonephric tubule (msn. tubule) and the mesonephric duct (msn. duct). The enlargement of the square is shown in F and J (HE staining). In both, F (white arrow) and J (black arrow) micrometastasis is indicated (high magnifications in G and H). In (E) HE staining of a transversal section approximately 24 μm rostral to the anterior start of the liver is shown and overlayed with the fluorescent image. A larger cell mass is indicated by an arrow. The same cell mass is indicated in I in which also a liver metastasis is seen. The cell mass is shown in high magnification in K (fluorescent picture) and in L (HE staining). Scales shown are A and E 1 mm, B-D, F-H and J-L 10 μm and in I: 500 μm.

**Figure 5:**
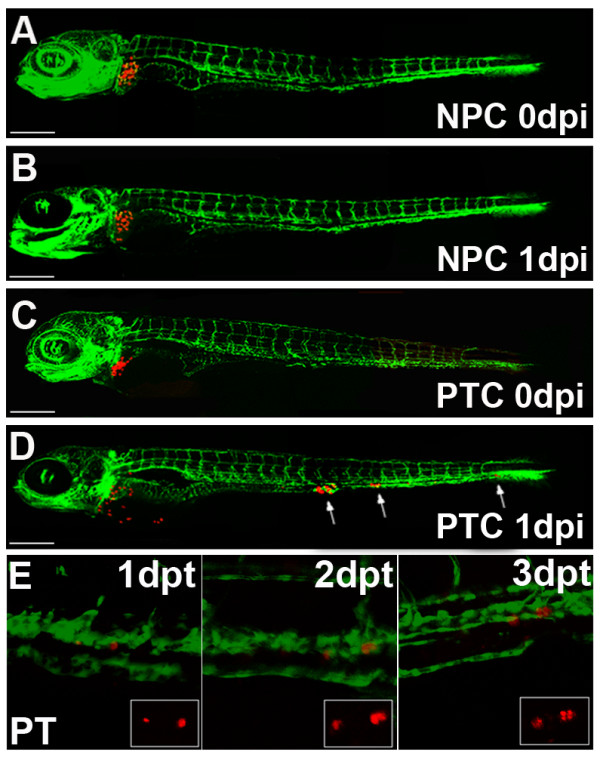
**Implantation of primary human tumour cells into the zebrafish liver**. (A-D) Organotopic implantation of primary tumour cells into the liver of larvae of Fli-1 zebrafish. Representative examples of zebrafish at 5 days of development injected with primary normal pancreatic cells (NPC) and with primary dissociated pancreatic tumour cells (A and C, respectively) are shown. The same fish are depicted at 1 day post injection (1 dpi). While normal pancreatic cells remained at the site of implantation in the liver (B), pancreatic tumour cells invaded the embryo and formed distant metastases, indicated with arrows (D). Scales indicated are: A-D 300 μm. Individuals were followed for up to 7 dpi and untransformed control cells never invaded the host embryos and remained in the liver for the entire observation period (data not shown). Image E shows an example of proliferating tumour cells of a transplanted pancreatic tumour fragment on consecutive days. The single cell on the right seen at 1 day post transplantation is divided into two daughter cells on 2 dpt and four cells are visible at 3 dpt. Dual colour laser scanning confocal images of the Fli-1 zebrafish are shown and in the smaller insert the red fluorescence of the CM-Dil labelled tumour cells can be seen.

In total, pancreatic tumours of four different patients were analysed. Three had carcinomas of the pancreas head and one had an adenocarcinoma of the ampulla vateri with infiltration of the pancreas (cancer grades and pTMN stages for all tumours are shown in table 1). On average 59.8% (SD +/- 5.2) of transplanted pancreatic tumour fragments showed invasion and migration in the developing zebrafish (table 1 and Fig. 3). Evaluation criteria for invasion and migration were that at least 5 cells had to be identified outside of the yolk and detectable within the developing zebrafish (table 1). Development of micrometastases was assessed by the presence of daughter cells at 3 dpt. The results are listed in table 1 and in Fig 4 examples of micrometastases in different tissues detected in sections of 5 dpf zebrafish are shown at high resolution (Fig. 4B, C, D, G, H, K and 4L). An example showing proliferating pancreatic tumour cells and the initial formation of a micrometastasis is given in Fig 5(E). Cell division is further indicated by PCNA immunostaining of invasive tumour cells in the zebrafish embryo (see Additional file 8). After 5 dpf embryos fall under strict local animal experiment regulations, therefore most embryos were not followed for longer periods. It is likely that the number of micrometastases would still increase over time.

**Table 1:** Tumour xenotransplantations into 2 day old zebrafish

	Tumour Transplants (grade and pTMN stage)	Number transplants (survivors)	Migration [presence of micro-metastases at 3 dpi]			total >5 [%]	Control Transplants	Number transplants (survivors)	Migration [presence of micro-metastases at 3 dpi]			total >5 [%]	p-value
			<5	5 to 20	>20				<5	5 to 20	>20		

1	Pancreas G2; *T3; N1 (13/14) R1, M1*	80 (64)	0	20 [4]	25 [20]	70.3%	Normal Pancreas	80 (73)	0	0	0	0	6.3 × 10^-5^

2	Pancreas G2; *pT3, pN1, pM1*	60 (42)	2 [0]	18 [4]	6 [4]	57.1%	Chronic Pancreatitis	60 (50)	0	0	0	0	1.1 × 10^-5^

3	Pancreas *G2; pT1, pN1 (1/18) pM1*	80 (46)	0	18 [5]	8 [7]	56.5%	Chronic Pancreatitis	80 (59)	0	0	0	0	1.9 × 10^-4^

4	Pancreas G3; *pT3, pN1 mi (1/11) pM1*	80 (65)	0	24 [6]	12 [9]	55.4%	Chronic Pancreatitis	80 (58)	0	0	0	0	5.4 × 10^-5^

5	Colon G2; *pT3 pN1 (2/14) LN metas.*	80 (66)	0	15 [7]	14 [10]	43.9%	Normal Tissue	80 (72)	0	0	0	0	0.9 × 10^-6^

6	Stomach G2: *pT3 pN2 (11/18 LK)*	80 (62)	0	23 [6]	11 [9]	54.8%	Normal Tissue	80 (68)	0	0	0	0	2.9 × 10^-5^

7	Stomach G2; ypT2b, ypN0 (0/20)	80 (48)	0	18 [5]	7 [6]	52.3%	Normal Tissue	80 (51)	0	0	0	0	1 × 10^-6^

	**Primary tumour cell injections**	**Number** **transplants** **(survivors)**	**Migration** **[presence of micro-metastases at 3 dpi]**			**total >5 [%]**	**Primary control cell injections**	**Number** **transplants** **(survivors)**	**Migration** **[presence of micro-metastases at 3 dpi]**			**total >5 [%]**	**p-value**

			<5	5 to 20	>20				<5	5 to 20	>20		

1	Pancreas	80 (63)	3 [0]	38 [10]	4 [4]	66.6%	Normal Pancreas	80 (69)	0	0	0	0	9.1 × 10^-5^

2	Pancreas	80 (66)	0	23 [7]	3 [2]	39.4%	Chronic Pancreatitis	80 72	2 [0]	0	0	0	7.6 × 10^-5^

3	Pancreas	80 (63)	0	22 [7]	6 [5]	44.4%	Chronic Pancreatitis	80 (61)	1 [0]	0	0	0	4 × 10^-4^

4	Pancreas	80 (63)	0	23 [6]	5 [4]	44.4%	Chronic Pancreatitis	80 (63)	1 [0]	0	0	0	5 × 10^-4^

5	Colon	80 (65)	0	24 [7]	5 [4]	44.4	Normal Tissue	80 (59)	1	0	0	0	2.7 × 10^-5^

6	Stomach	80 (68)	0	24 [6]	0	35.3	Normal Tissue	80 (69)	0	0	0	0	6.8 × 10^-6^

Tumours as numbered in the table: ^1^Pancreas head carcinoma; ^2^Well differentiated ductal adenocarcinoma of the Pancreas head; ^3^Moderately differentiated ductal adenocarcinoma of the Pancreas head; ^4^Poorly differentiated adenocarcinoma of the ampulla vateri with infiltration of the pancreas; ^5^Adenocarcinoma of the colon; ^6^Moderately differentiated adenocarcinoma of the stomach; ^7^Moderately differentiated tubular adenocarcinoma of the stomach. For the tumour cell injections in the lower half of the table numbers 1–6 indicate the tumour sources of the dissociated cells. There is a significant difference between all tumours and controls and all p-values of a student t-test were below 0.001. Tumour grading and classification is according to histopathological examination. Development of micrometastases was assessed by the presence of daughter cells at 3 days post transplantation (dpt).

Invasive cells were found for pancreatic tumours in blood islands, caudal fin, caudal vein, gut, heart, hindbrain, intestine, liver, mesonephric duct, mesonephric tubule, mandible, operculum, pericardium, somites, swim bladder, for the colon tumour in caudal vein, gut, heart, intestine, liver, pericardium; and for the stomach tumours in the caudal vein, gill arches, heart, intestine, liver, mandible, otic cup, pericardium;

Similar to preferences observed for PaTu-T cells, tumour cells of implanted gastrointestinal tumor tissue fragments had a tendency to invade and home in to organs of the gastrointestinal tract. Micrometastasis were often observed in the liver, the gut and the intestine. Homing in of these cells in muscle tissue and the formation of micrometastasis was rarely observed. Although we observed qualitative differences for the preferential homing in of the two cell lines tested (PaTu-T and TGF-β treated EpRAS cells), a future study, with a larger cohort of tumour specimen and tumour types is necessary to determine tumour specific preferences.

Fig 3 shows examples of fish embryos directly after transplantation (A, B) and at 1- and 3-dpt (C-G). Cell invasion and micrometastasis formation of pancreatic tumour cells is clearly detectable 24 h after transplantation (E). Similar results were also obtained for transplanted tissue fragments of a colon adenocarcinoma (43.9% invasion and migration) and two moderately differentiated adenocarcinomas of the stomach (average 53.5%, SD +/- 1.2) (p-values in all tumour experiments were < 0.001). As a control for the tumours we used colon and gastric mucosal fragments or peritumoural, non-transformed tissue of the respective tissue explants from the same patients. In the pancreas the non-transformed tissue controls mostly showed histological manifestations of chronic pancreatitis and only one was considered as having a normal pancreatic histology.

Histological sections of control pancreas and of pancreatic cancer tissue of human patients are shown in Fig 3(J, K). In all control transplantations of chronic pancreatitis specimen and of fragments of normal pancreas, colon and stomach tissue cellular invasion and migration was never observed (see Additional file 1 and Fig. 3D, F). In addition, we also tested the metastatic behaviour of a benign tumour. Tissue fragments of adenomateous colonic polyps (0,4 cm and 1 cm) were investigated, which had not yet invaded through the lamina muscularis mucosae. No metastasis was observed for either of them in the zebrafish embryo (Additional file 1). Comparable results were seen when cells were dissociated from tumour or control tissue samples prior to their injection into the zebrafish. All four primary pancreatic tumour cells showed cellular invasion and migration with an average of 48.8% (SD +/- 9.0) (see Additional file 1). In the case of dissociated primary colon and stomach tumour cells 44.4% and 35.3% of cell injections, respectively, resulted in cellular invasion and migration (see Additional file 1).

Xenotransplantation experiments of tumour fragments as well as the injection of isolated primary tumour cells allowed to discriminate between non-invasive chronic pancreatitis and infiltrating pancreatic adenocarcinoma. In real-time, we show an example of pancreatic tumour cells in the zebrafish 1 day after a pancreatic cancer fragment was transplanted (see Additional file 9). This movie shows circulating tumour cells and tumour cells which have extravasated into the perivascular tissue of a 3 day old zebrafish. A moving primary human tumour cell passing through the caudal vein and into an intersegmental vessel is also visible. In a supplemental movie we show how a pancreatic tumour cell is slowly traversing through the caudal vein of a 3 dpt Tg(fli1:eGFP) zebrafish (see Additional file 10). In Fig 4 histological sections of zebrafish embryos transplanted with a pancreatic human tumour are shown at 3 dpt. Examples of micrometastases in the liver (B, C and D) and near the mesonephric duct (G and H) are shown at higher magnifications. A cell mass is further shown in K and L.

### Organotopic transplantation of primary human tumour cells in the fish liver and the effects of protease inhibitors on the invasiveness of implanted tumour cells and tumour fragments

Some xenograft models that have been established in the mouse involve the orthotopic transplantation of specific human tumours and tumour cells [29,30]. Surgical orthotopic implantations (SOI) of tumour cells or of resected primary tumour fragments into immune deficient mice have proven useful for studying their growth and metastatic potential [31].

Here we transplanted freshly dissociated primary pancreatic tumour cells and normal pancreatic control cells into the liver of zebrafish larvae. For these experiments, we used the Tg(fli1:eGFP) transgenic zebrafish line (with the advantage of a fluorescent vasculature), which allows an exact localization and injection into the highly vascularised liver.

Primary cells of control pancreatic tissue, when transplanted into the liver, remained at the site of the injection and did not invade the developing zebrafish nor did they enter the blood circulation (Fig. 5). In contrast, primary tumour cells invaded the neighbouring tissue, entered the circulation and migrated and homed in at distant tissues and organs (Fig. 5).

Furthermore we investigated in our zebrafish xenotransplantation model the effects of protease inhibitors on the invasiveness of implanted tumour cells and tumour fragments. Two different protease inhibitors were able to inhibit the invasiveness of tumour cells and of a primary pancreatic tumour (see Additional file 1).

## Discussion

Analysis of tumour metastasis in an in vivo model depends on intrinsic tumour cell properties, host factors and the experimental techniques used. The engraftment of human neoplasms in the mouse normally requires the use of nude (athymic) or severe combined immune deficient (SCID) mice that are T-and B-cell deficient. In these animal models further attention has to be paid to the site of implantation, as host factors may differ between tissues and organs. Nude mice also have an upregulated innate immunity and elevated numbers of natural killer cells and tumoricidal macrophages, which may limit tumour growth or even prevent metastasis. Efficacy of pharmacological and toxicological studies in murine xenograft models normally use tumour growth, body weight loss and mortality as parameters of toxicity. These studies are cumbersome, time consuming and drug activity against xenografts does not always correlate with its clinical activity [32].

In our study we established the zebrafish as a robust *in vivo *model for investigating invasiveness and metastatic behaviour of human primary tumours. It is known that early zebrafish embryos do not reject xenotransplanted human cells [13-15], whereas 1 month old zebrafish already need to be immune suppressed [11].

The early embryos and larvae used here did not reject the primary tumour xenografts, most likely due to the fact that their immune system is not fully developed. It has been observed that while lymphopoiesis and lymphoid-organogenesis are initiated at the middle to late embryo period, they remained in their rudimentary and immature form throughout the early larval stages. The major maturation events leading to immune competence occur between 2 and 4 weeks post fertilisation (wpf), coinciding with the larval to juvenile transitory phase [33].

The observed metastasis in an animal model primarily should reflect the intrinsic metastatic ability of the tumour cells, but may depend to some extent also on the experimental system. Other experimental animal systems have demonstrated that only a small subset of metastatic cells (approximately 2%) survive and grow at secondary sites [34]. The significantly higher percentage of micrometastases observed using fish embryos may in part reflect the absence of the humoral immune response and/or other selective pressures on tumors cells which would lead to tumor cell death following extravastaion into secondary organs.

The transparency of the fish embryo enables an investigation of fluorescently labelled tumour cells in real time and at high resolution. The unique availability of transgenic zebrafish without a functional vasculature [17] further allowed us to show that the metastatic spread of tumour cells in zebrafish embryos involves the vascular system. Even the very early steps of invasion, circulation of tumour cells in blood vessels, colonization at secondary organ sites and metastasis formation can be observed this way-something which to date cannot be investigated in established mouse tumour models. Advantages of the model system such as good accessibility, easy handling, low costs and short incubation times make it a promising system for future functional studies in primary tumours.

The experiments described here provide the basis for the future development of a screening methodology of drugs, which inhibit invasion and metastasis of human tumours. Recently, adult zebrafish with an almost entirely transparent body have been described, as a novel tool for *in vivo *transplantation analysis [35]. These will be of interest for additional comparative analysis of metastasis formation of primary tumours in the immune competent animal.

## Conclusion

We demonstrate here the applicability of the zebrafish embryo as an *in vivo *model for the analysis of metastatic behaviour of human tumour cells, including resection specimen from human tissue. High resolution imaging of live zebrafish has and will further assist in better understanding the underlying mechanisms of cancer cell invasion and metastasis formation. Advantages of the model system such as good accessibility, easy handling, low costs and rapidness are unparalleled by other vertebrate organisms and make it a promising system for future functional studies in primary tumours. The advantages of the "short term" zebrafish embryo model could nicely complement established "longer term" tumour models, e.g. mouse models, and may be a valuable and efficient tool to evaluate novel therapeutic strategies for cancer.

## Competing interests statement

The authors declare that they have no competing interests.

## Authors' contributions

IJM, FUW, CN, LIP, CDH, MML and CPB did experimental work and helped with writing the manuscript. CPB did project planning and wrote the manuscript. All authors read and approved the final manuscript.

## Pre-publication history

The pre-publication history for this paper can be accessed here:

http://www.biomedcentral.com/1471-2407/9/128/prepub

## Supplementary Material

Additional file 1 **Supplemental Tables**. Tables of all experiments including tumour transplantations and injections of primary tumor cells, control tissues, EpRas and EpRasTGF-β cells, PaTu-S and PaTu-T cells,Protease inhibitor treated cells and benign tumours (Colon Polyps). Click here for file

Additional file 2 **EpRas cells**. 3D reconstruction of EpRas cells in the yolk at 3dpi (20x magnification). Click here for file

Additional file 3 **TGF-β treated EpRas cells**. 3D reconstruction of EpRas TGF-β treated cells in the dorsal aorta at 3dpi (20x magnification). Click here for file

Additional file 4 **Time lapse movie of implanted TGF-β treated EpRas cells**. Supplemental Movie 1: Time lapse movie (rate: 1 frame/minute) showing EpRas TGF-β cells which have invaded the zebrafish body, have translocated into the vasculature and have colonized distant sites in the 5 day old larvae. Some cells are visible in the blood stream whereas others have extravasated from the vasculature.
Click here for file

Additional file 5 **Wound healing *in vitro *migration assay ("scratch assay")**. Cell-based *in vitro *migration assay for PaTu-S and PaTu-T cells. Click here for file

Additional file 6 **Lack of circulation in cloche^-/- ^mutants**. Real time movie of control fish and cloche^-/- ^fish at 3 dpf (1 dpi). The movie shows a control fish with a beating heart and with visible circulating blood cells. The second part of the movie illustrates a cloche^-/- ^fish with a beating heart. No circulation is observable in the mutant fish; the third part of the movie is a higher magnification of a control fish in the area of the blood islands and tail, showing cells circulating in the vasculature e.g. caudal vein, dorsal aorta and intersegmental vessels. The last part of the movie shows a higher magnification of a cloche^-/- ^fish in the same area of the blood islands as the control. In contrast to the control zebrafish, the cloche^-/- ^fish does not possess a functional vascular system and shows no sign of circulation. Click here for file

Additional file 7 **No metastatic behaviour of implanted tumour cells in cloche mutant embryos**. PaTu-T cells injected into control and cloche^-^/^- ^embryos. Click here for file

Additional file 8 **PCNA staining**. Whole mount immunofluorescence of a pancreatic tumour transplanted embryo. Click here for file

Additional file 9 **Circulation of pancreatic tumour cells**. Real-time movie of pancreatic tumour cells in the zebrafish 1 day after a pancreatic cancer fragment was transplanted. Circulating tumour cells and tumour cells which have extravasated into the perivascular tissue of a 3 day old zebrafish are visible. A moving primary human tumour cell passing through the caudal vein and into an intersegmental vessel is also visible. Click here for file

Additional file 10 **Tumour cell in the vasculature**. Real-time movie of a pancreatic cancer cell slowly traversing through the vasculature (the caudal vein) of a Tg(fli1:eGFP) zebrafish embryo. Click here for file
