# Supplementary material for: Metastatic behaviour of primary human tumours in a zebrafish xenotransplantation model
Source: BMC Cancer. 2009 Apr 28;9:128. doi: 10.1186/1471-2407-9-128 (PMC2697170; doi:10.1186/1471-2407-9-128)
Supplement: Additional file 1 — Supplemental Tables. Tables of all experiments including tumour transplantations and injections of primary tumor cells, control tissues, EpRas and EpRasTGF-β cells, PaTu-S and PaTu-T cells,Protease inhibitor treated cells and benign tumours (Colon Polyps). [file 1471-2407-9-128-S1.doc]

**Tables for all experiments**

(including Tumor Transplants and injections of primary tumor cells, control tissues, EpRas and EpRasTGF-β cells, PaTu-S and PaTu-T cells,Protease inhibitor treated cells and benign tumours (Colon Polyps).

-----------------------------------------------------------------------------------------------------Tumor Transplants and injections of primary tumor cells plus controls

| **Patient 1 (11.10.2007) Pancreatic Tumor** | | | | | | | |  |  |  |  |  |  |
| --- | --- | --- | --- | --- | --- | --- | --- | --- | --- | --- | --- | --- | --- |
|  |  |  |  |  |  |  |  |  |  |  |  |  |  |
|  |  | **Tumor tissue** | | |  |  | **Normal Pancreas tissue** | | |  |  |  |  |
|  |  |  |  |  |  |  |  |  |  |  |  |  |  |
| Day | Transplanted | Alive | Migration | | | **total >5** | Day | Transplanted | Alive | Migration | | | **total >5** |
|  |  |  | <5 | 5 to 20 | >20 |  |  |  |  | <5 | 5 to 20 | >20 |  |
| 0 | 80 | 80 | 0 | 0 | 0 | 0 | 0 | 80 | 80 | 0 | 0 | 0 | 0 |
| 1 |  | **64** | 0 | 20 | 25 | 45 | 1 |  | **73** | 0 | 0 | 0 | 0 |
| 2 |  | 60 | 0 | 16 | 28 | 44 | 2 |  | 68 | 0 | 0 | 0 | 0 |
| 3 |  | 52 | 0 | 16 | 20 | 36 | 3 |  | 67 | 0 | 0 | 0 | 0 |
|  |  |  |  |  |  | **45 (70.3 %)** |  |  |  |  |  |  | **0 (0%)** |
|  |  |  |  |  |  |  |  |  |  |  | p=6.34921E-05 | | |
|  | **Tumor (dissociated primary cells)** | | | | |  | **Normal Pancreas (dissociated primary cells)** | | | | | | |
|  |  |  |  |  |  |  |  | |  |  |  |  |  |
| Day | Injected | Alive | Migration | | | **total >5** | Day | Injected | Alive | Migration | | | **total >5** |
|  |  |  | <5 | 5 to 20 | >20 |  |  |  |  | <5 | 5 to 20 | >20 |  |
| 0 | 80 | 80 | 0 | 0 | 0 | 0 | 0 | 80 | 80 | 0 | 0 | 0 | 0 |
| 1 |  | **72** | 3 | 38 | 4 | 42 | 1 |  | **69** | 0 | 0 | 0 | 0 |
| 2 |  | 71 | 4 | 44 | 4 | 48 | 2 |  | 62 | 0 | 0 | 0 | 0 |
| 3 |  | 67 | 5 | 40 | 3 | 43 | 3 |  | 59 | 0 | 0 | 0 | 0 |
|  |  |  |  |  |  | **48 (66.6%)** |  |  |  |  |  |  | **0 (0%)** |
|  |  |  |  |  |  |  |  |  |  |  | p=9.10712E-06 | | |
|  |  |  |  |  |  |  |  |  |  |  |  |  |  |
|  |  |  |  |  |  |  |  |  |  |  |  |  |  |
| **Patient 2 (14.10.2007) Pancreatic Tumor** | | | | | | | |  |  |  |  |  |  |
|  |  |  |  |  |  |  |  |  |  |  |  |  |  |
|  |  | **Tumor tissue** | | |  |  | **Chronic Pancreatitis tissue** | | | |  |  |  |
|  |  |  |  |  |  |  |  |  |  |  |  |  |  |
| Day | Transplanted | Alive | Migration | | | **total >5** | Day | Transplanted | Alive | Migration | | | **total >5** |
|  |  |  | <5 | 5 to 20 | >20 |  |  |  |  | <5 | 5 to 20 | >20 |  |
| 0 | 60 | 60 | 0 | 0 | 0 | 0 | 0 | 60 | 60 | 0 | 0 | 0 | 0 |
| 1 |  | **42** | 2 | 16 | 5 | 21 | 1 |  | **50** | 0 | 0 | 0 | 0 |
| 2 |  | 40 | 0 | 18 | 6 | 24 | 2 |  | 48 | 0 | 0 | 0 | 0 |
| 3 |  | 40 | 0 | 16 | 8 | 24 | 3 |  | 47 | 0 | 0 | 0 | 0 |
|  |  |  |  |  |  | **24 (57.1 %)** |  |  |  |  |  |  | **0 (0%)** |
|  |  |  |  |  |  |  |  |  |  |  | p=1.05866E-05 | | |
|  | **Tumor (dissociated primary cells)** | | | | |  | **Chronic Pancreatitis (dissociated primary cells)** | | | | | | |
|  |  |  |  |  |  |  |  | |  |  |  |  |  |
| Day | Injected | Alive | Migration | | | **total >5** | Day | Injected | Alive | Migration | | | **total >5** |
|  |  |  | <5 | 5 to 20 | >20 |  |  |  |  | <5 | 5 to 20 | >20 |  |
| 0 | 80 | 80 | 0 | 0 | 0 | 0 | 0 | 80 | 80 | 0 | 0 | 0 | 0 |
| 1 |  | **66** | 0 | 23 | 3 | 26 | 1 |  | **72** | 2 | 0 | 0 | 0 |
| 2 |  | 61 | 0 | 22 | 4 | 26 | 2 |  | 72 | 0 | 0 | 0 | 0 |
| 3 |  | 56 | 0 | 20 | 3 | 23 | 3 |  | 69 | 0 | 0 | 0 | 0 |
|  |  |  |  |  |  | **26 (39.4%)** |  |  |  |  |  |  | **0 (0%)** |
|  |  |  |  |  |  |  |  |  |  |  | p=7.59876E-06 | | |
|  |  |  |  |  |  |  |  |  |  |  |  |  |  |
|  |  |  |  |  |  |  |  |  |  |  |  |  |  |
| **Patient 3 (18 and 19.10.2007) Pancreatic Tumor** | | | | | | | | |  |  |  |  |  |
|  |  |  |  |  |  |  |  |  |  |  |  |  |  |
|  |  | **Tumor tissue** | | |  |  | **Chronic Pancreatitis tissue** | | | |  |  |  |
|  |  |  |  |  |  |  |  |  |  |  |  |  |  |
| Day | Transplanted | Alive | Migration | | | **total >5** | Day | Transplanted | Alive | Migration | | | **total >5** |
|  |  |  | <5 | 5 to 20 | >20 |  |  |  |  | <5 | 5 to 20 | >20 |  |
| 0 | 80 | 80 | 0 | 0 | 0 | 0 | 0 | 80 | 80 | 0 | 0 | 0 | 0 |
| 1 |  | 50 | 0 | 14 | 5 | 19 | 1 |  | **59** | 0 | 0 | 0 | 0 |
| 2 |  | **46** | 0 | 18 | 8 | 26 | 2 |  | 46 | 0 | 0 | 0 | 0 |
| 3 |  | 40 | 0 | 16 | 8 | 24 | 3 |  | 41 | 0 | 0 | 0 | 0 |
|  |  |  |  |  |  | **26 (56.5 %)** |  |  |  |  |  |  | **0 (0%)** |
|  |  |  |  |  |  |  |  |  |  |  | p=0.000190766 | | |
|  | **Tumor (dissociated primary cells)** | | | | |  | **Chronic Pancreatitis (dissociated primary cells)** | | | | | | |
|  |  |  |  |  |  |  |  | |  |  |  |  |  |
| Day | Injected | Alive | Migration | | | **total >5** | Day | Injected | Alive | Migration | | | **total >5** |
|  |  |  | <5 | 5 to 20 | >20 |  |  |  |  | <5 | 5 to 20 | >20 |  |
| 0 | 80 | 80 | 0 | 0 | 0 | 0 | 0 | 80 | 80 | 0 | 0 | 0 | 0 |
| 1 |  | **63** | 0 | 22 | 6 | 28 | 1 |  | **61** | 1 | 0 | 0 | 0 |
| 2 |  | 57 | 0 | 16 | 5 | 21 | 2 |  | 59 | 0 | 0 | 0 | 0 |
| 3 |  | 54 | 0 | 16 | 3 | 19 | 3 |  | 53 | 0 | 0 | 0 | 0 |
|  |  |  |  |  |  | **28 (44.4%)** |  |  |  |  |  |  | **0 (0%)** |
|  |  |  |  |  |  |  |  |  |  |  | p=0.000481985 | | |
|  |  |  |  |  |  |  |  |  |  |  |  |  |  |
|  |  |  |  |  |  |  |  |  |  |  |  |  |  |
| **Patient 4 (23.10.2007) Pancreatic Tumor** | | | | | | | |  |  |  |  |  |  |
|  |  |  |  |  |  |  |  |  |  |  |  |  |  |
|  |  | **Tumor tissue** | | |  |  | **Chronic Pancreatitis tissue** | | | |  |  |  |
|  |  |  |  |  |  |  |  |  |  |  |  |  |  |
| Day | Transplanted | Alive | Migration | | | **total >5** | Day | Transplanted | Alive | Migration | | | **total >5** |
|  |  |  | <5 | 5 to 20 | >20 |  |  |  |  | <5 | 5 to 20 | >20 |  |
| 0 | 80 | 80 | 0 | 0 | 0 | 0 | 0 | 80 | 80 | 0 | 0 | 0 | 0 |
| 1 |  | **65** | 0 | 24 | 12 | 36 | 1 |  | **58** | 0 | 0 | 0 | 0 |
| 2 |  | 59 | 0 | 20 | 15 | 35 | 2 |  | 48 | 0 | 0 | 0 | 0 |
| 3 |  | 52 | 0 | 17 | 12 | 29 | 3 |  | 42 | 0 | 0 | 0 | 0 |
|  |  |  |  |  |  | **36 (55.4 %)** |  |  |  |  |  |  | **0 (0%)** |
|  |  |  |  |  |  |  |  |  |  |  | p=5.3915E-05 | | |
|  | **Tumor (dissociated primary cells)** | | | | |  | **Chronic Pancreatitis (dissociated primary cells)** | | | | | | |
|  |  |  |  |  |  |  |  | |  |  |  |  |  |
| Day | Injected | Alive | Migration | | | **total >5** | Day | Injected | Alive | Migration | | | **total >5** |
|  |  |  | <5 | 5 to 20 | >20 |  |  |  |  | <5 | 5 to 20 | >20 |  |
| 0 | 80 | 80 | 0 | 0 | 0 | 0 | 0 | 80 | 80 | 0 | 0 | 0 | 0 |
| 1 |  | **58** | 0 | 23 | 5 | 28 | 1 |  | **63** | 1 | 0 | 0 | 0 |
| 2 |  | 49 | 0 | 14 | 4 | 18 | 2 |  | 59 | 0 | 0 | 0 | 0 |
| 3 |  | 0 | 0 | 16 | 3 | 19 | 3 |  | 50 | 0 | 0 | 0 | 0 |
|  |  |  |  |  |  | **28 (48.2%)** |  |  |  |  |  |  | **0 (0%)** |
|  |  |  |  |  |  |  |  |  |  |  | p=0.000481985 | | |
| **Patient 5 (9.11.2007) Colon Tumor** | | | | | | |  |  |  |  |  |  |  |
|  |  |  |  |  |  |  |  |  |  |  |  |  |  |
|  |  | Tumor tissue | | |  |  | **Normal Colon tissue** | | |  |  |  |  |
|  |  |  |  |  |  |  |  |  |  |  |  |  |  |
| Day | Transplanted | Alive | Migration | | | **total >5** | Day | Transplanted | Alive | Migration | | | **total >5** |
|  |  |  | <5 | 5 to 20 | >20 |  |  |  |  | <5 | 5 to 20 | >20 |  |
| 0 | 80 | 80 | 0 | 0 | 0 | 0 | 0 | 80 | 80 | 0 | 0 | 0 | 0 |
| 1 |  | **66** | 0 | 15 | 14 | 29 | 1 |  | **72** | 0 | 0 | 0 | 0 |
| 2 |  | 60 | 0 | 14 | 15 | 29 | 2 |  | 62 | 0 | 0 | 0 | 0 |
| 3 |  | 55 | 0 | 13 | 14 | 27 | 3 |  | 42 | 0 | 0 | 0 | 0 |
|  |  |  |  |  |  | **29 (43.9%)** |  |  |  |  |  |  | **0 (0%)** |
|  |  |  |  |  |  |  |  |  |  |  | p=9.16145E-07 | | |
|  | **Tumor (dissociated primary cells)** | | | | |  | **Normal Colon (dissociated primary cells)** | | | | | |  |
|  |  |  |  |  |  |  |  | |  |  |  |  |  |
| Day | Injected | Alive | Migration | | | **total >5** | Day | Injected | Alive | Migration | | | **total >5** |
|  |  |  | <5 | 5 to 20 | >20 |  |  |  |  | <5 | 5 to 20 | >20 |  |
| 0 | 80 | 80 | 0 | 0 | 0 | 0 | 0 | 80 | 80 | 0 | 0 | 0 | 0 |
| 1 |  | **68** | 0 | 24 | 5 | 29 | 1 |  | **59** | 1 | 0 | 0 | 0 |
| 2 |  | 59 | 0 | 22 | 4 | 26 | 2 |  | 57 | 0 | 0 | 0 | 0 |
| 3 |  | 42 | 0 | 20 | 4 | 24 | 3 |  | 54 | 0 | 0 | 0 | 0 |
|  |  |  |  |  |  | **28 (41.2%)** |  |  |  |  |  |  | **0 (0%)** |
|  |  |  |  |  |  |  |  |  |  |  | p=2.72494E-05 | | |
|  |  |  |  |  |  |  |  |  |  |  |  |  |  |
|  |  |  |  |  |  |  |  |  |  |  |  |  |  |
| **Patient 6 (20.11.2007) Stomach Tumor** | | | | | | | |  |  |  |  |  |  |
|  |  |  |  |  |  |  |  |  |  |  |  |  |  |
|  |  | **Tumor tissue** | | |  |  | **Normal Stomach tissue** | | |  |  |  |  |
|  |  |  |  |  |  |  |  |  |  |  |  |  |  |
| Day | Transplanted | Alive | Migration | | | **total >5** | Day | Transplanted | Alive | Migration | | | **total >5** |
|  |  |  | <5 | 5 to 20 | >20 |  |  |  |  | <5 | 5 to 20 | >20 |  |
| 0 | 80 | 80 | 0 | 0 | 0 | 0 | 0 | 80 | 80 | 0 | 0 | 0 | 0 |
| 1 |  | **62** | 0 | 23 | 11 | 34 | 1 |  | **68** | 0 | 0 | 0 | 0 |
| 2 |  | 55 | 0 | 21 | 11 | 32 | 2 |  | 62 | 0 | 0 | 0 | 0 |
| 3 |  | 49 | 0 | 18 | 10 | 28 | 3 |  | 52 | 0 | 0 | 0 | 0 |
|  |  |  |  |  |  | **34 (54.8%)** |  |  |  |  |  |  | **0 (0%)** |
|  |  |  |  |  |  |  |  |  |  |  | p=2.94989E-05 | | |
|  | Tumor (dissociated primary cells) | | | | |  | **Normal Stomach (dissociated primary cells)** | | | | | | |
|  |  |  |  |  |  |  |  | |  |  |  |  |  |
| Day | Injected | Alive | Migration | | | **total >5** | Day | Injected | Alive | Migration | | | **total >5** |
|  |  |  | <5 | 5 to 20 | >20 |  |  |  |  | <5 | 5 to 20 | >20 |  |
| 0 | 80 | 80 | 0 | 0 | 0 | 0 | 0 | 80 | 80 | 0 | 0 | 0 | 0 |
| 1 |  | **68** | 0 | 24 | 0 | 24 | 1 |  | **69** | 0 | 0 | 0 | 0 |
| 2 |  | 59 | 0 | 22 | 1 | 23 | 2 |  | 63 | 0 | 0 | 0 | 0 |
| 3 |  | 42 | 0 | 20 | 1 | 21 | 3 |  | 56 | 0 | 0 | 0 | 0 |
|  |  |  |  |  |  | **24 (35.3%)** |  |  |  |  |  |  | **0 (0%)** |
|  |  |  |  |  |  |  |  |  |  |  | p=6.8063E-06 | | |
|  |  |  |  |  |  |  |  |  |  |  |  |  |  |
|  |  |  |  |  |  |  |  |  |  |  |  |  |  |
| **Patient 7 Stomach Tumor** | | | | | |  |  |  |  |  |  |  |  |
|  |  |  |  |  |  |  |  |  |  |  |  |  |  |
|  |  | **Tumor tissue** | | |  |  | **Normal Stomach tissue** | | |  |  |  |  |
|  |  |  |  |  |  |  |  |  |  |  |  |  |  |
| Day | Transplanted | Alive | Migration | | | **total >5** | Day | Transplanted | Alive | Migration | | | **total >5** |
|  |  |  | <5 | 5 to 20 | >20 |  |  |  |  | <5 | 5 to 20 | >20 |  |
| 0 | 80 | 80 | 0 | 0 | 0 | 0 | 0 | 80 | 80 | 0 | 0 | 0 | 0 |
| 1 |  | **48** | 0 | 18 | 7 | 25 | 1 |  | **51** | 0 | 0 | 0 | 0 |
| 2 |  | 44 | 0 | 17 | 8 | 25 | 2 |  | 47 | 0 | 0 | 0 | 0 |
| 3 |  | 41 | 0 | 17 | 7 | 24 | 3 |  | 44 | 0 | 0 | 0 | 0 |
|  |  |  |  |  |  | **25 (52.1%)** |  |  |  |  |  |  | **0 (0%)** |
|  |  |  |  |  |  |  |  |  |  |  | p=9.99231E-08 | | |
| No cells tested | |  |  |  |  |  |  |  |  |  |  |  |  |
|  |  |  |  |  |  |  |  |  |  |  |  |  |  |
| t-test: | |  |  |  |  |  |  |  |  |  |  |  |  |
| All p-values for tumors versus control tissue and tumor cells versus control cells were below 0.0002 | | | | | | | | | | | | | |

------------------------------------------------------------------------------------------------------------

Injections of EpRas and EpRasTGFb cells:

| **Experiment1** | |  |  |  |  |  |  |  |  |  |  |  |  |
| --- | --- | --- | --- | --- | --- | --- | --- | --- | --- | --- | --- | --- | --- |
|  |  | **EpRas** | |  |  |  | **EpRasTGFb** | |  |  |  |  |  |
|  |  |  |  |  |  |  |  |  |  |  |  |  |  |
| Day | Injected | Alive | Migration | | | **total >5** | Day | Injected | Alive | Migration | | | **total >5** |
|  |  |  | <5 | 5 to 20 | >20 |  |  |  |  | <5 | 5 to 20 | >20 |  |
| 0 | 80 | 80 | 0 | 0 | 0 | 0 | 0 | 138 | 138 | 0 |  | 0 | 0 |
| 1 |  | **61** | 2 | 1 | 0 | 1 | 1 |  | **101** | 8 | 38 | 9 | 47 |
| 2 |  | 55 | 0 | 0 | 0 | 0 | 2 |  | 92 | 5 | 36 | 8 | 44 |
| 3 |  | 48 | 0 | 0 | 0 | 0 | 3 |  | 90 | 5 | 35 | 8 | 43 |
|  |  |  |  |  |  | **1 (1.6 %)** |  |  |  |  |  |  | **47 (46.5%)** |
|  |  |  |  |  |  |  |  |  |  |  | p=1.86932E-06 | | |
| **Experiment2** | |  |  |  |  |  |  |  |  |  |  |  |  |
|  |  | **EpRas** | |  |  |  | **EpRasTGFb** | |  |  |  |  |  |
|  |  |  |  |  |  |  |  | |  |  |  |  |  |
| Day | Injected | Alive | Migration | | | **total >5** | Day | Injected | Alive | Migration | | | **total >5** |
|  |  |  | <5 | 5 to 20 | >20 |  |  |  |  | <5 | 5 to 20 | >20 |  |
| 0 | 80 | 80 | 0 | 0 | 0 | 0 | 0 | 80 | 80 | 0 | 0 | 0 | 0 |
| 1 |  | **66** | 0 | 0 | 0 | 0 | 1 |  | **72** | 12 | 32 | 4 | 36 |
| 2 |  | 60 | 0 | 0 | 0 | 0 | 2 |  | 65 | 12 | 30 | 5 | 35 |
| 3 |  | 57 | 0 | 0 | 0 | 0 | 3 |  | 59 | 10 | 28 | 5 | 33 |
|  |  |  |  |  |  | **0 (0%)** |  |  |  |  |  |  | **36 (50%)** |
|  |  |  |  |  |  |  |  |  |  |  | p=1.25116E-06 | | |
| **Experiment3** | |  |  |  |  |  |  |  |  |  |  |  |  |
|  |  | **EpRas** | |  |  |  | **EpRasTGFb** | |  |  |  |  |  |
|  |  |  |  |  |  |  |  |  |  |  |  |  |  |
| Day | Injected | Alive | Migration | | | **total >5** | Day | Injected | Alive | Migration | | | **total >5** |
|  |  |  | <5 | 5 to 20 | >20 |  |  |  |  | <5 | 5 to 20 | >20 |  |
| 0 | 80 | 80 | 0 | 0 | 0 | 0 | 0 | 80 | 80 | 0 | 0 | 0 | 0 |
| 1 |  | **70** | 0 | 0 | 0 | 0 | 1 |  | **76** | 11 | 29 | 3 | 32 |
| 2 |  | 63 | 0 | 0 | 0 | 0 | 2 |  | 68 | 9 | 30 | 3 | 33 |
| 3 |  | 54 | 0 | 0 | 0 | 0 | 3 |  | 65 | 7 | 29 | 2 | 31 |
|  |  |  |  |  |  | **0 (0 %)** |  |  |  |  |  |  | **33 (43.4%)** |
|  |  |  |  |  |  |  |  |  |  |  | p=3.17203E-07 | | |

------------------------------------------------------------------------------------------------------------

Consecutive injections of **PaTu-S and PaTu-T** in the same embryo

| **Experiment1** | |  |  |  |  |  |  |  |  |  |
| --- | --- | --- | --- | --- | --- | --- | --- | --- | --- | --- |
|  |  | **PaTu-S and PaTu-T** | | | |  |  |  |  |  |
|  |  |  |  |  |  |  |  |  |  |  |
| Day | Injected | Alive | Migration (Green) | | | **total >5** | Migration (Red) | | | **total >5** |
|  |  |  | <5 | 5 to 20 | >20 |  | <5 | 5 to 20 | >20 |  |
| 0 | 80 | 80 | 0 | 0 | 0 | 0 | 0 | 0 | 0 | 0 |
| 1 |  | **62** | 0 | 0 | 0 | 0 | 4 | 12 | 2 | 14 |
| 2 |  | 61 | 0 | 0 | 0 | 0 | 4 | 11 | 3 | 14 |
| 3 |  | 52 | 0 | 0 | 0 | 0 | 3 | 11 | 2 | 13 |
|  |  |  |  |  |  | **0 (0 %)** |  |  |  | **14 (22.6%)** |
|  |  |  |  |  |  |  |  | p=1.05746E-06 | | |
|  |  |  |  |  |  |  |  |  |  |  |
| **Experiment2** | |  |  |  |  |  |  |  |  |  |
|  |  | **PaTu-S and PaTu-T** | | | |  |  |  |  |  |
|  |  |  |  |  |  |  |  |  |  |  |
| Day | Injected | Alive | Migration (Green) | | | **total >5** | Migration (Red) | | | **total >5** |
|  |  |  | <5 | 5 to 20 | >20 |  | <5 | 5 to 20 | >20 |  |
| 0 | 80 | 80 | 0 | 0 | 0 | 0 | 0 | 0 | 0 | 0 |
| 1 |  | **60** | 2 | 0 | 0 | 0 | 3 | 14 | 1 | 15 |
| 2 |  | 53 | 0 | 0 | 0 | 0 | 3 | 14 | 1 | 15 |
| 3 |  | 48 | 0 | 0 | 0 | 0 | 2 | 12 | 1 | 13 |
|  |  |  |  |  |  | **0 (0%)** |  |  |  | **15 (25%)** |
|  |  |  |  |  |  |  |  | p=4.15681E-06 | | |
|  |  |  |  |  |  |  |  |  |  |  |
| **Experiment3** | |  |  |  |  |  |  |  |  |  |
|  |  | **PaTu-S and PaTu-T** | | | |  |  |  |  |  |
|  |  |  |  |  |  |  |  |  |  |  |
| Day | Injected | Alive | Migration (Green) | | | **total >5** | Migration (Red) | | | **total >5** |
|  |  |  | <5 | 5 to 20 | >20 |  | <5 | 5 to 20 | >20 |  |
| 0 | 80 | 80 | 0 | 0 | 0 | 0 | 0 | 0 | 0 | 0 |
| 1 |  | **57** | 0 | 0 | 0 | 0 | 6 | 19 | 2 | 21 |
| 2 |  | 47 | 0 | 0 | 0 | 0 | 5 | 16 | 1 | 17 |
| 3 |  | 41 | 0 | 0 | 0 | 0 | 5 | 12 | 1 | 13 |
|  |  |  |  |  |  | **0 (0 %)** |  |  |  | **21 (36.8%)** |
|  |  |  |  |  |  |  |  | p=0.000907184 | | |

------------------------------------------------------------------------------------------------------------

**Benign tumours (Colon Polyps):**

The size of the two benign polyps was 0.4 cm (A) and 1 cm (B), respectively. Infiltration of the lamina muscularis mucosae had not been detected, indicating that they are both benign tumours.

| **Colon Polyp A** | | | | | | |
| --- | --- | --- | --- | --- | --- | --- |
| Day |  | | Migration | | | |
|  | Injected | Alive | <5 | 5 to 20 | >20 | **total >5** |
| 0 | 80 | 0 | 0 | 0 | 0 | 0 |
| 1 |  | 54 | 0 | 0 | 0 | 0 |
| 2 |  | 48 | 0 | 0 | 0 | 0 |
| 3 |  | 42 | 0 | 0 | 0 | 0 |
|  |  |  |  |  |  | **0 (0%)** |

| **Colon Polyp B** | | | | | | |
| --- | --- | --- | --- | --- | --- | --- |
| Day |  | | Migration | | | |
|  | Injected | Alive | <5 | 5 to 20 | >20 | **total >5** |
| 0 | 80 | 80 | 0 | 0 | 0 | 0 |
| 1 |  | 52 | 0 | 0 | 0 | 0 |
| 2 |  | 46 | 0 | 0 | 0 | 0 |
| 3 |  | 45 | 0 | 0 | 0 | 0 |
|  |  |  |  |  |  | **0 (0%)** |

------------------------------------------------------------------------------------------------------------

**Protease inhibitors: effects on invasion of PaTu-T cells and of transplanted pancreatic tumour fragments.**

Two different protease inhibitorswere tested: MMP-2/MMP-9 inhibitor V from Calbiochem (Cat.No.444285) and NNGH from Biomol (BML-205). Both were used at a final concentration of 5 μM. Cells or tumour fragments were treated overnight with the inhibitors and implanted the next day. Controls were treated with DMSO.

**Experiment1**

| **MMP-2/MMP-9 (PaTu-T cells)** | | | | | | |
| --- | --- | --- | --- | --- | --- | --- |
| Day |  | | Migration | | | |
|  | Injected | Alive | <5 | 5 to 20 | >20 | **total >5** |
| 0 | 80 | 80 | 0 | 0 | 0 | 0 |
| 1 |  | 70 | 2 | 0 | 0 | 0 |
| 2 |  | 62 | 1 | 0 | 0 | 0 |
| 3 |  | 54 | 1 | 0 | 0 | 0 |
|  |  |  |  |  |  | **0 (0%)** |

| **NNGH (PaTu-T cells)** | | | | | | |
| --- | --- | --- | --- | --- | --- | --- |
| Day |  | | Migration | | | |
|  | Injected | Alive | <5 | 5 to 20 | >20 | **total >5** |
| 0 | 80 | 80 | 0 | 0 | 0 | 0 |
| 1 |  | 60 | 0 | 0 | 0 | 0 |
| 2 |  | 58 | 0 | 0 | 0 | 0 |
| 3 |  | 54 | 0 | 1 | 0 | 0 |
|  |  |  |  |  |  | **1 (1.8 %)** |

| **DMSO Control (PaTu-T cells)** | | | | | | |
| --- | --- | --- | --- | --- | --- | --- |
| Day |  | | Migration | | | |
|  | Injected | Alive | <5 | 5 to 20 | >20 | **total >5** |
| 0 | 80 | 80 | 0 | 0 | 0 | 0 |
| 1 |  | 69 | 2 | 18 | 2 | 20 |
| 2 |  | 63 | 1 | 17 | 2 | 19 |
| 3 |  | 58 | 1 | 16 | 1 | 17 |
|  |  |  |  |  |  | **20 (26.1%)** |

**Experiment2**

| **MMP-2/MMP-9 (PaTu-T cells)** | | | | | | |
| --- | --- | --- | --- | --- | --- | --- |
| Day |  | | Migration | | | |
|  | Injected | Alive | <5 | 5 to 20 | >20 | **total >5** |
| 0 | 80 | 80 | 0 | 0 | 0 | 0 |
| 1 |  | 66 | 1 | 0 | 0 | 0 |
| 2 |  | 61 | 2 | 0 | 0 | 0 |
| 3 |  | 58 | 1 | 0 | 0 | 0 |
|  |  |  |  |  |  | **0 (0%)** |

| **NNGH (PaTu-T cells)** | | | | | | |
| --- | --- | --- | --- | --- | --- | --- |
| Day |  | | Migration | | | |
|  | Injected | Alive | <5 | 5 to 20 | >20 | **total >5** |
| 0 | 80 | 80 | 0 | 0 | 0 | 0 |
| 1 |  | 72 | 0 | 0 | 0 | 0 |
| 2 |  | 66 | 1 | 0 | 0 | 0 |
| 3 |  | 57 | 0 | 0 | 0 | 0 |
|  |  |  |  |  |  | **0 (0%)** |

| **DMSO Control (PaTu-T cells)** | | | | | | |
| --- | --- | --- | --- | --- | --- | --- |
| Day |  | | Migration | | | |
|  | Injected | Alive | <5 | 5 to 20 | >20 | **total >5** |
| 0 | 80 | 80 | 0 | 0 | 0 | 0 |
| 1 |  | 63 | 2 | 17 | 1 | 18 |
| 2 |  | 61 | 1 | 17 | 1 | 18 |
| 3 |  | 59 | 1 | 16 | 1 | 17 |
|  |  |  |  |  |  | **18 (27.1%)** |

**Tumour Experiment**

Adenocarcinoma of the pancreas, which originated from an intraductal papillary mucinous neoplasm (IPMN), but already showed malignancy as documented by the presence of lymph node metastases.

| **MMP-2/MMP-9 (Pancreatic Tumour)** | | | | | | |
| --- | --- | --- | --- | --- | --- | --- |
| Day |  | | Migration | | | |
|  | Injected | Alive | <5 | 5 to 20 | >20 | **total >5** |
| 0 | 80 | 80 | 0 | 0 | 0 | 0 |
| 1 |  | 59 | 1 | 0 | 0 | 0 |
| 2 |  | 54 | 1 | 0 | 0 | 0 |
| 3 |  | 50 | 1 | 0 | 0 | 0 |
|  |  |  |  |  |  | **0 (0%)** |

| **NNGH (Pancreatic Tumour)** | | | | | | |
| --- | --- | --- | --- | --- | --- | --- |
| Day |  | | Migration | | | |
|  | Injected | Alive | <5 | 5 to 20 | >20 | **total >5** |
| 0 | 80 | 80 | 0 | 0 | 0 | 0 |
| 1 |  | 58 | 1 | 0 | 0 | 0 |
| 2 |  | 58 | 0 | 0 | 0 | 0 |
| 3 |  | 53 | 0 | 0 | 0 | 0 |
|  |  |  |  |  |  | **0 (0%)** |

| **DMSO Control (Pancreatic Tumour)** | | | | | | |
| --- | --- | --- | --- | --- | --- | --- |
| Day |  | | Migration | | | |
|  | Injected | Alive | <5 | 5 to 20 | >20 | **total >5** |
| 0 | 80 | 80 | 0 | 0 | 0 | 0 |
| 1 |  | 56 | 2 | 21 | 6 | 27 |
| 2 |  | 51 | 2 | 19 | 5 | 24 |
| 3 |  | 50 | 1 | 16 | 4 | 20 |
|  |  |  |  |  |  | **27 (48.2%)** |

**Cloche mutant and control Experiments**

Cloche-/- zebrafish and siblings with no phenotype (with a functional vasculature and circulation) and additional control Tg:fli1/eGFP fish were injected (at 2 dpf) with PaTu-T cells and followed over time.

| **Experiment1** | |  |  |  |  |  |  |  |  |  |  |  |  |
| --- | --- | --- | --- | --- | --- | --- | --- | --- | --- | --- | --- | --- | --- |
| **PaTu-T in cloche-/-** | | | | | | | **PaTu-T in siblings with no phenotype** | | | | | | |
|  |  |  |  |  |  |  |  |  |  |  |  |  |  |
| Day | Injected | Alive | Migration | | | **total >5** | Day | Injected | Alive | Migration | | | **total >5** |
|  |  |  | <5 | 5 to 20 | >20 |  |  |  |  | <5 | 5 to 20 | >20 |  |
| 0 | 53 | 53 | 0 | 0 | 0 | 0 | 0 | 80 | 80 | 0 | 0 | 0 | 0 |
| 1 |  | **50** | 0 | 0 | 0 | 0 | 1 |  | **75** | 10 | 27 | 5 | 32 |
| 2 |  | 49 | 0 | 0 | 0 | 0 | 2 |  | 70 | 9 | 28 | 4 | 32 |
| 3 |  | 47 | 0 | 0 | 0 | 0 | 3 |  | 67 | 8 | 27 | 4 | 31 |
|  |  |  |  |  |  | **0 (0 %)** |  |  |  |  |  |  | **32 (42.7%)** |
|  |  |  |  |  |  |  |  |  |  |  |  | | |

| **Experiment1** |  |  |  |  |  |  |
| --- | --- | --- | --- | --- | --- | --- |
| **PaTu-T in control Tg:fli1/eGFP fish** | | | | | | |
|  |  |  |  |  |  |  |
| Day | Injected | Alive | Migration | | | **total >5** |
|  |  |  | <5 | 5 to 20 | >20 |  |
| 0 | 80 | 80 | 0 | 0 | 0 | 0 |
| 1 |  | **73** | 6 | 24 | 4 | 28 |
| 2 |  | 67 | 6 | 25 | 3 | 28 |
| 3 |  | 62 | 5 | 24 | 2 | 26 |
|  |  |  |  |  |  | **28 (38.4%)** |

| **Experiment2** | |  |  |  |  |  |  |  |  |  |  |  |  |
| --- | --- | --- | --- | --- | --- | --- | --- | --- | --- | --- | --- | --- | --- |
| **PaTu-T in cloche-/-** | | | | | | | **PaTu-T in siblings with no phenotype** | | | | | | |
|  |  |  |  |  |  |  |  |  |  |  |  |  |  |
| Day | Injected | Alive | Migration | | | **total >5** | Day | Injected | Alive | Migration | | | **total >5** |
|  |  |  | <5 | 5 to 20 | >20 |  |  |  |  | <5 | 5 to 20 | >20 |  |
| 0 | 63 | 63 | 0 | 0 | 0 | 0 | 0 | 80 | 80 | 0 | 0 | 0 | 0 |
| 1 |  | **61** | 0 | 0 | 0 | 0 | 1 |  | **76** | 10 | 29 | 8 | 37 |
| 2 |  | 59 | 0 | 0 | 0 | 0 | 2 |  | 70 | 7 | 30 | 5 | 35 |
| 3 |  | 56 | 0 | 0 | 0 | 0 | 3 |  | 60 | 4 | 27 | 2 | 29 |
|  |  |  |  |  |  | **0 (0 %)** |  |  |  |  |  |  | **37 (48.7%)** |
|  |  |  |  |  |  |  |  |  |  |  |  | | |

| **Experiment2** |  |  |  |  |  |  |
| --- | --- | --- | --- | --- | --- | --- |
| **PaTu-T in control Tg:fli1/eGFP fish** | | | | | | |
|  |  |  |  |  |  |  |
| Day | Injected | Alive | Migration | | | **total >5** |
|  |  |  | <5 | 5 to 20 | >20 |  |
| 0 | 80 | 80 | 0 | 0 | 0 | 0 |
| 1 |  | **72** | 7 | 24 | 6 | 30 |
| 2 |  | 69 | 9 | 26 | 4 | 30 |
| 3 |  | 67 | 8 | 26 | 4 | 30 |
|  |  |  |  |  |  | **30 (41.7%)** |
