# Supplementary material for: Metastatic behaviour of primary human tumours in a zebrafish xenotransplantation model
Source: BMC Cancer. 2009 Apr 28;9:128. doi: 10.1186/1471-2407-9-128 (PMC2697170; doi:10.1186/1471-2407-9-128)
Supplement: Additional file 5 — Wound healing in vitro migration assay ("scratch assay"). Cell-based in vitro migration assay for PaTu-S and PaTu-T cells. [file 1471-2407-9-128-S5.doc]

For each sample 5 readings of distance were measured at 0, 6 and 15 h.

Gap closure is calculated as proportion (mean +/- Std. dev.) of the initial Gap width.
