# Supplementary figures and images for: Metastatic behaviour of primary human tumours in a zebrafish xenotransplantation model
Source: BMC Cancer. 2009 Apr 28;9:128. doi: 10.1186/1471-2407-9-128 (PMC2697170; doi:10.1186/1471-2407-9-128)

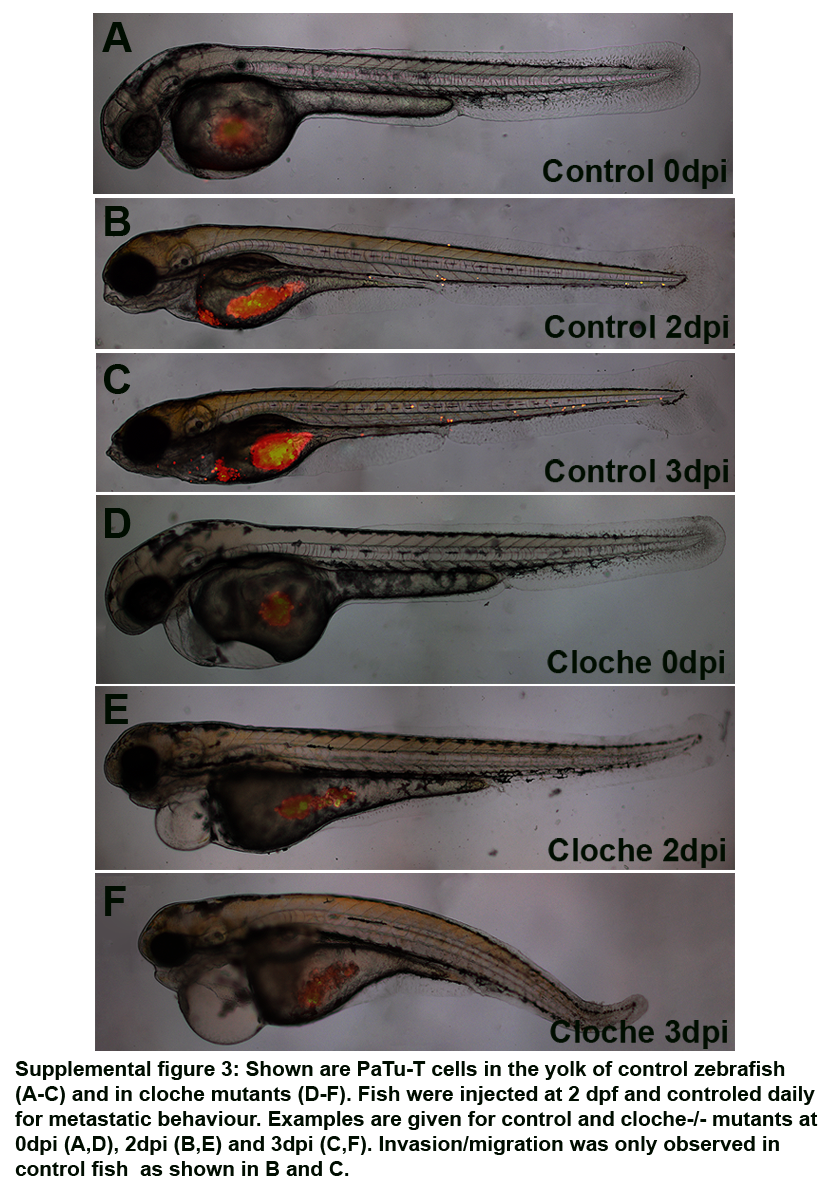

Supplement: Additional file 7 — No metastatic behaviour of implanted tumour cells in cloche mutant embryos. PaTu-T cells injected into control and cloche-/- embryos. [file 1471-2407-9-128-S7.tiff]

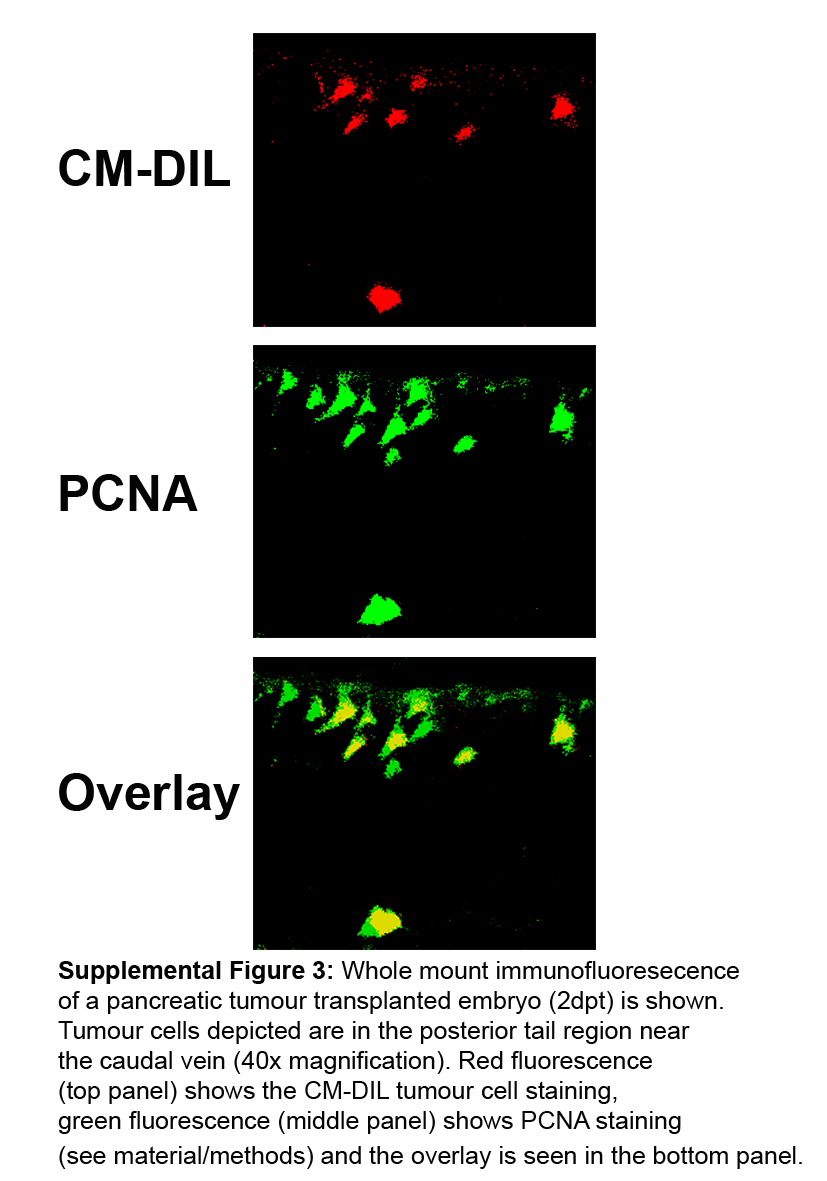

Supplement: Additional file 8 — PCNA staining. Whole mount immunofluorescence of a pancreatic tumour transplanted embryo. [file 1471-2407-9-128-S8.tiff]
